# The Effect of Selective Occlusal Adjustment on the Disclusion Time Reduction and Symmetry of Occlusal Contacts of the Own Dentition Using Digital Occlusion Analysis in Patients with Temporomandibular Disorders

**DOI:** 10.3390/jcm14197007

**Published:** 2025-10-03

**Authors:** Wojciech Maga, Martyna Schönborn, Małgorzata Pihut

**Affiliations:** 1 Prosthodontic and Orthodontic Department, Dental Institute, Medical College, Jagiellonian University, 4 Montelupich Str., 31-155 Krakow, Poland; malgorzata.pihut@uj.edu.pl; 2 Department of Angiology, Faculty of Medicine, Jagiellonian University Medical College, 30-663 Krakow, Poland; martyna.schoenborn@uj.edu.pl

**Keywords:** temporomandibular disorders, T-Scan, disclusion time reduction, bite forces, digital analysis of occlusion, occlusal corrective treatment, digital occlusion analysis

## Abstract

**Background/Objectives**: Occlusal disturbances occurring during central occlusion, mandibular movements and mastication may contribute to the development of temporomandibular disorders (TMDs). To reduce the disclusion time (DT) in all mandibular contacts, a procedure known as enameloplasty can be applied. The aim of this study was to evaluate the effect of occlusion-correcting treatments on disclusion time reduction, determination of the center of force, and the distribution of masticatory forces on the right and left side, through digital occlusal analysis in patients with TMD. **Methods**: The single-centered, prospective study including 106 patients with TMD after 6 months of prosthetic treatment. Digital occlusal analysis was performed before and after the enameloplasty to assess occlusion time, disclusion time and symmetry of occlusal contacts. **Results**: Selective enameloplasty significantly reduced disclusion time in the whole study population (0.8 vs. 0.4 s; *p* < 0.001), with greater improvement observed in patients with inappropriate Center of Force (COF) and premature occlusal contacts. **Conclusions**: Digital occlusal analysis-guided selective enameloplasty effectively reduces disclusion time and can improve occlusal parameters in TMD patients.

## 1. Introduction

The forces produced by the activity of the masticatory muscles are transferred to the teeth through occlusal contacts [1]. Physiological occlusion is characterized by well-defined conditions and points of contact of teeth in opposing arches, and maintaining occlusal anatomy and harmony greatly depends on the duration of these contacts [2,3]. Therefore, an abnormal occlusal pattern of the teeth may become the cause of numerous adverse changes in the masticatory system and may lead to temporomandibular disorders (TMDs). TMDs are characterized by pain and noises in the temporomandibular joint, frequent temporal headaches, neck pain, ear pain, chewing fatigue, clenching and grinding habits, morning facial muscle pain and stiffness, and restricted mouth opening [4]. There are many reasons for these abnormalities, ranging from congenital defects in the dentition, the consequences of failing to replace missing teeth, to the many iatrogenic errors of dental treatment [5]. Occlusal disturbances and malocclusions have been increasingly recognized as important predisposing factors for functional imbalance within the stomatognathic system. In particular, mandibular crowding has been associated with altered occlusal contacts, abnormal force distribution, and impaired functional stability, which may contribute to the development or persistence of temporomandibular disorders (TMDs) [6]. Recent scoping reviews emphasize that accurate diagnosis and appropriate management of such occlusal conditions are essential not only for maintaining dental alignment, but also for preserving functional harmony of the masticatory system. During excursive movements, occlusal disturbances play a significant role and are considered contributing factors in the development of these disorders. Hyperactivity of the masticatory muscles may also lead to overload on restorations, resulting in complications such as strong tooth sensitivity, fractures of restorations or abutment teeth, increased tooth mobility, and post-restorative temporomandibular issues [7]. There is a well-established relationship between the duration of posterior tooth contacts during central occlusion, mandibular excursive movements, and the level of activity in the masticatory muscles [8,9].

In the digital analysis of occlusion, important parameters are the so-called occlusion time (OT) and discussion time (DT). OT is closely linked to the patient’s occlusal contact pattern and is regarded as a reliable indicator of occlusion, while DT may connect tooth contacts with muscle activity [10]. DT is defined as the time needed for a patient to move from full tooth contact and shift in one direction—right, left, or forward—so that all molars and premolars separate, leaving only the canines and/or incisors in contact [8]. A DT longer than 0.4 s indicates that occlusal forces are applied for an extended period. Sustained contact under greater force can cause alteration of the occlusal surfaces [3].

Correction or reconstruction of physiological occlusion is one of the most important tasks of modern dental prosthodontics. It may be achieved by proper reduction in prolonged disclusion time through a procedure called immediate complete anterior guidance development (ICAGD) enameloplasty. This procedure is often integrated with complementary treatment modalities (such as occlusal splints or physiotherapy), providing significant support in the overall treatment of patients with TMD [11,12]. ICAGD is an approach for occlusal therapy for establishing immediate posterior disclusion in all mandibular movements, prior to any habitual closure adjustment. It was verified that once DT is properly reduced to less than 0.5 s, it is a permanent occlusal change that allows the retention of proper muscle function. The technique of this procedure includes two steps, one of which is to reduce the DT to 0.4 s [8,13]. The disclusion time reduction (DTR) procedure aids in managing occlusal forces by decreasing their duration, strength, and intensity on the tooth surfaces, and has been shown to help preserve stable occlusal anatomy [7].

This study aimed to evaluate the effect of occlusion-correcting treatments on the disclusion time reduction in a group of patients undergoing prosthetic treatment for TMD based on the result of digital T-Scan analysis. Furthermore, the impact of bite force asymmetry between the right and left sides, assessment of the center of the force (occlusal force vector resultant obtained during T-Scan analysis), and the presence of premature contacts on disclusion and occlusion time were analyzed.

## 2. Materials and Methods

### 2.1. Study Design and Population

We performed a single-centered and prospective study including patients from the Department of Prosthetics and Orthodontics between January 2024 and April 2025. All participants underwent a clinical examination according to the Axis I DC/TMD (International Network for Orofacial Pain and Related Disorders Methodology; 2018) and it was used in the diagnosis of dysfunction. After the examination, patients were qualified for prosthetic treatment due to symptoms of TMD (pain of the masticatory muscles, limited opening of the mouth, popping and clicking in the temporomandibular joints) as well as inclusion and exclusion criteria. Firstly, patients underwent a 6 month treatment program for TMD, in accordance with current medical guidelines. They used hard relaxation splints for approximately 21 h per day. In addition, they attended physiotherapy sessions at least once per week and performed home exercises prescribed by the doctor. Furthermore, patients were advised to implement behavioral modifications, including improvements in sleep hygiene, stress management in daily life, and the avoidance of deleterious habits and parafunctional activities that could exacerbate their condition. After 6 months of therapy, patients were qualified for enameloplasty. Digital occlusal analysis was performed before and after the procedure to correct the configuration of the teeth in the opposing dental arches, assess the percentage of occlusal contacts between the right and left sides, their location, and contact force, as well as the disclusion and occlusion time.

All T-Scan recordings and selective enameloplasty procedures were carried out by a single experienced operator following a strict and standardized protocol, which minimized potential variability. The intraobserver reliability was calculated based on preliminary experiments using the reliability statistics of the interclass correlation (ICC 1, 2). The ICC coefficient was 0.945 (0.910–0.986 CI) for disclusion time.

The study was conducted in accordance with the Declaration of Helsinki and approved by the Institutional Ethics Committee of Jagiellonian University (protocol code 1072.6120.271.2022).

### 2.2. The Inclusion and Exclusion Criteria

Participants were eligible for inclusion if they met the following conditions:

- Aged 20–50 years old.
- Complete natural dentition comprising at least 28 teeth.
- Presence of chronic muscle-related myofascial pain symptoms consistent with temporomandibular dysfunction.
- Diagnosis of muscle disorders classified as Group I according to the DC/TMD criteria.
- Diagnosis of disk displacement disorders corresponding to Group II of the DC/TMD classification.
- Near-normal occlusal relationships, characterized by preserved contacts between opposing premolars and molars during lateral excursions to both the right and left sides.
- Six months (minimum) of prosthetic treatment of the temporomandibular joint
- Qualification for enameloplasty.

Patients were excluded from the study if any of the following conditions were present:

- Temporomandibular joint pain (arthralgia) or degenerative joint disorders, including osteoarthritis and osteoarthrosis, classified as Group III according to the DC/TMD.
- Previous occlusal adjustment therapy.

### 2.3. Dysfunction Analysis

Axis I of the DC/TMD was used in the diagnosis of the dysfunction. This system of classification was developed according to the biopsychosocial model of pain, incorporating an Axis I evaluation of physical factors with standardized diagnostic criteria, and an Axis II evaluation addressing psychosocial aspects and pain-related disability [14,15]. The diagnostic system is divided into three groups: (I) muscle disorders, (II) disk displacements, and (III) arthralgia, osteoarthritis, and osteoarthrosis. In this study, only patients with disorders classified as Group I or II were included.

### 2.4. Digital Occlusal Analysis

**Figure 1:**
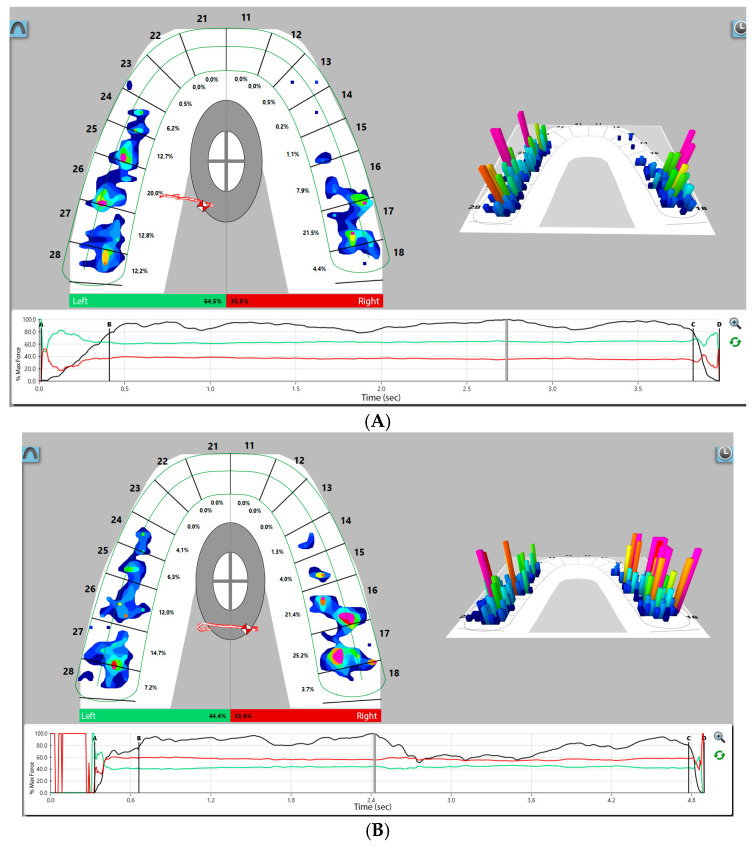
Same participant’s T-Scan record. (**A**) Two-dimensional and three-dimensional force views before ICAGD enameloplasty; (**B**) 2D and 3D force views show short disclusion time achieved with ICAGD enameloplasty.

Measurements were taken using the T-Scan Novus device. The occlusal sensor is designed to register dynamic occlusal interactions by allowing the patient to clench or perform masticatory movements on its surface. During functional mandibular activity, the sensor records real-time variations in relative tooth-to-tooth contact forces. As opposing dentition establishes occlusal contact, compression of the upper and lower sensor layers occurs, resulting in resistance alterations within each activated sensel. The magnitude of these resistance changes is directly proportional to the applied occlusal load: greater occlusal forces induce larger resistance shifts, whereas lower forces produce proportionally smaller changes. The T-Scan software provides a multi-colored, two- and three-dimensional graphical visualization of dynamic occlusal parameters, enabling the identification of occlusal disturbances and their associated non-physiological muscular responses (Figure 1A,B). The digital occlusal analysis encompassed quantification of the distribution of occlusal contacts between the right and left sides, the spatial localization of these contacts, their relative intensity represented by a color-coded force scale, and the measurement of disclusion time. The indication for occlusal correction was a disclusion time exceeding 0.5 s.

During the examination using the T-Scan III Novus system, the patient was positioned in a dental chair with the head aligned in a maximally upright position to prevent distal displacement of the mandible, which could otherwise result in inaccurate readings. A new, single-use sensor was placed intraorally, ensuring that the plastic marker contacted the interincisal space between the maxillary central incisors, and that the plane of the sensor was as parallel as possible to the occlusal plane. Additionally, colored markers were used on the sensor to indicate the interdental spaces between the lateral incisors and canines in the maxilla, as well as between the premolars. The patient was instructed to bite down on the sensor with maximum force, following the initiation of recording via the control head. The occlusion was maintained for approximately 10 s, after which the patient was asked to open their mouth. During the second examination, the patient was instructed to perform lateral mandibular excursions while maintaining intercuspation on the sensor, continuing until posterior disclusion occurred, in order to evaluate the disclusion time. Each examination was performed twice per patient, following prior calibration of the sensor’s sensitivity.

### 2.5. Enameloplasty

All patients who had completed a 6 month TMD therapy program were enrolled and underwent occlusal equilibration through the ICAGD method described by Kerstein and Farrell. The procedure involved computer-assisted ICAGD enameloplasty, performed with simultaneous T-Scan recordings—before and after the enameloplasty. The primary objective of this procedure is to reduce the anterior disclusion time to <0.4 s, and the secondary objective is to reduce the signs and symptoms [14]. Enameloplasty is a minimally invasive procedure involving controlled removal of hard dental tissues—most commonly cusp tips and portions of occlusal surfaces—to improve occlusal guidance and balance contacts. When performed within the limits of enamel, the procedure does not compromise tooth vitality, while providing significant functional benefits in terms of occlusal harmony. In this study, the procedure was performed using a high-speed handpiece with fine-grit diamond burs, under constant water cooling to minimize thermal damage. Adjustments were carried out in small, incremental steps and verified with digital occlusal analysis systems to ensure precise elimination of premature contacts. Following enamel reduction, the treated surfaces were polished to restore smoothness and reduce plaque retention.

### 2.6. Statistical Analysis

Absolute and percentage values were used to describe nominal variables, and their significance was assessed using the chi-square test. For variables not normally distributed, the median and interquartile range (IQR) were used. The Mann–Whitney U test was used to compare quantitative variables and assess the significance of treatment outcome between groups. The Wilcoxon signed-rank test was used to analyze the significance of treatment within a group. Spearman correlation was used for correlation analysis. All analyses were performed in IBM SPSS Statistics version 29.0.2.0 (IBM Corporation, Armonk, New York, NY, USA). The significance level was <0.05.

## 3. Results

### 3.1. Demographic and Clinical Characteristics of Subjects

The study included 106 patients with diagnosed TMD after a 6 month treatment program, qualified for enameloplasty. The mean age was 39 (33; 43) years, with a female predominance in gender distribution (73 [68.9%]). In the baseline digital occlusal analysis before treatment, 49 patients (46.2%) had an appropriate center of force; the mean disclusion time was 0.8 s, and the mean occlusion time was 0.35 s. Premature occlusal contacts were present in 75 subjects (70.8%). The whole baseline demographic and clinical characteristics are provided in Table 1.

Premature occlusal contacts revealed their frequent presence in the examined population.

### 3.2. Clinical Characteristics of Subjects Depending on the Center of the Force

We also analyzed the impact of the resultant forces acting in the oral cavity during biting on key clinical parameters, including those assessed by the T-Scan. The Center of Force (COF) represents the geometric point of convergence of all occlusal load vectors at a specific moment, indicating the spatial balance of occlusal contacts as detected by the T-Scan system. Patients with no appropriate COF were characterized by statistically significant prolonged disclusion time before and after the enameloplasty compared to subjects with appropriate center of the force (0.9 vs. 0.6 [s]; *p* < 0.001 and 0.4 vs. 0.3 [s]; *p* < 0.001). Interestingly, the difference in values before and after the procedure was significantly greater in patients with inappropriate COF (0.4 vs. 0.29 [s]; *p* < 0.001). The was no correlation between the center of the force and gender, age, site difference, maximal bite force, or occlusion time values. A comparison of clinical characteristics by the center of the force is provided in Table 2.

### 3.3. Disclusion Time Changes Before and After the Enameloplasty

**Figure 2:**
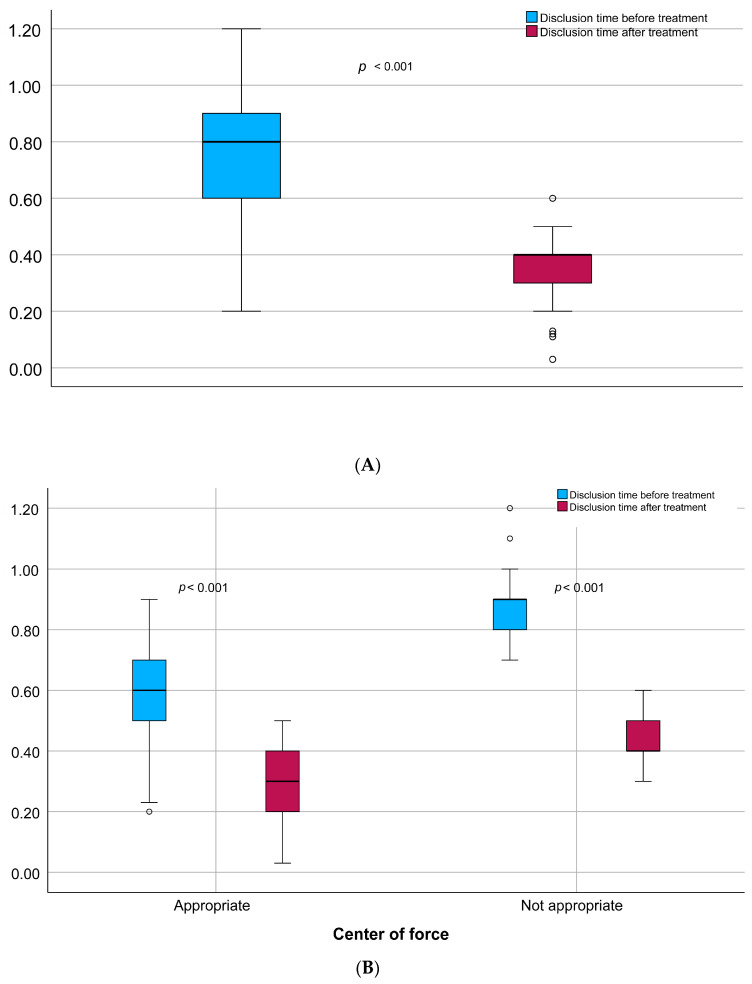
Disclusion time changes before and after treatment: (**A**) in the whole studied population, (**B**) depending on center of force.

In the whole observed population, mean DT was significantly lower after the procedure (0.4 vs. 0.8 [s], *p* < 0.001) (Figure 2A). In patients with malocclusion, the mean difference in disclusion time values after the procedure was greater. However, the values achieved remained significantly higher compared to those observed in individuals without inappropriate COF (0.4 vs. 0.3 [s]; *p* < 0.001) (Figure 2B).

**Figure 3:**
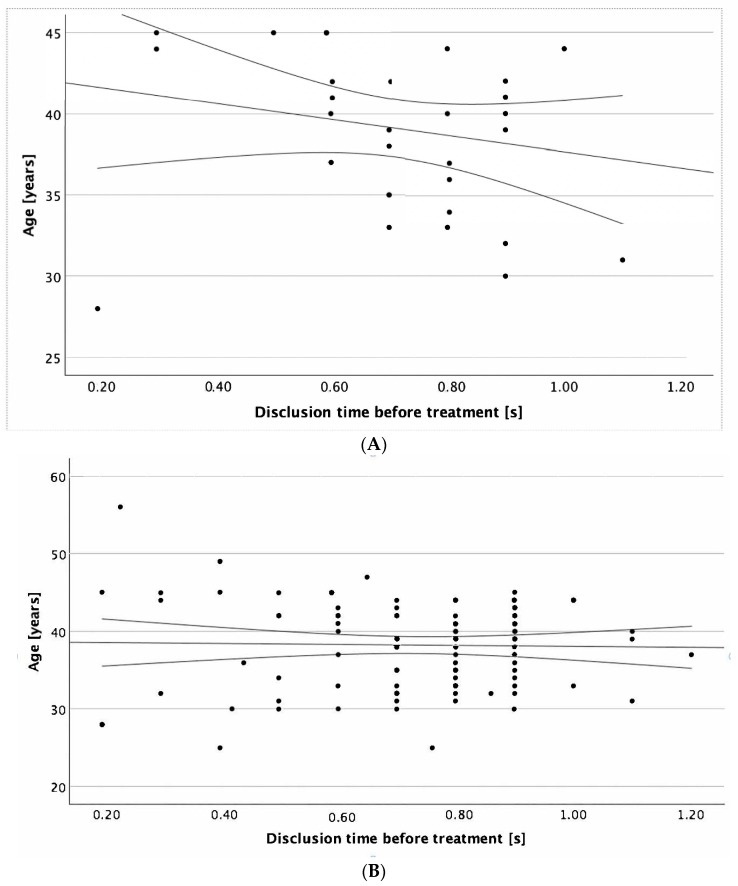
Disclusion time prior to treatment in correlation to the age of (**A**) the male patients (r = −0.356, *p* = 0.042), and (**B**) the patients regardless of gender (*p* = 0.89). Legend: Dots—the scatter of variables; central line—best fit line; external lines—confidence index.

Additionally, we conducted an analysis of factors that could potentially correlate with disclusion time values prior to performing enameloplasty. The results showed that only in the male group was age significantly correlated with DT values (r = −0.356, *p* = 0.042, Figure 3A).

## 4. Discussion

In our study, DTR was achieved in the whole analyzed population of patients with TMD, both in individuals with an initially disturbed occlusion and in those with a proper distribution of occlusal forces. These results are consistent with other studies on the use of the ICAGD enameloplasty method in improving the harmony of occlusal forces within the dentition and its positive impact on both DTR and symptom reduction [8,16,17,18]. In 1991, Kerstein et al. showed that ICAGD not only reduces disclusion time to <0.4 s but also significantly minimizes symptoms [8]. The study by Kerstein and Radke on 45 symptomatic TMD patients with myofascial pain showed that properly performed ICAGD may significantly reduce muscle activity level [4]. In a retrospective, five-year follow-up study on 30 patients with TMD after DTR therapy, the intensity and frequency of chronic pain significantly decreased while improving the overall, long-term quality of life [19]. Finally, the multicenter, randomized study by Thumati et al. on 100 subjects showed that ICAGD, compared to tooth polishing, alleviated pain, reduced symptom frequency, and emotional depression within 1 week, which continued for 6 months [10]. In the literature, there are also studies evaluating the influence of DTR on preventing the occlusal changes in posterior implant-supported prostheses. In one study with 12 patients, also assessing the association between DTR and crestal bone loss, ICAGD resulted in a significant decrease in OT and DT from 1.51 ± 0.6 s to 0.37 ± 0.06 s (*p* < 0.001). However, the mean crestal bone levels from day 1 to 6 months showed no significant changes [20].

Moreover, our study revealed a significant correlation in the male group between age and DT values. To the best of our knowledge, the available scientific literature does not contain direct studies describing the correlation between age and disclusion time. Nevertheless, the sample size may have been too small to draw definitive conclusions, and it would be necessary to test this hypothesis in a larger group of patients.

The contribution of our work is the evaluation of the Center of Force (COF), which enabled us to objectively determine the spatial balance of occlusal load distribution. The shift in the COF towards a more centralized and stable position after selective enameloplasty indicates improved occlusal symmetry, which has not been widely reported in previous studies on ICAGD. Furthermore, the assessment of premature occlusal contacts revealed their frequent presence in the examined population, underscoring their role as a potential etiological factor in TMD. Their successful elimination following guided enameloplasty not only shortened disclusion time but also contributed to a more physiologic occlusal scheme.

It should be emphasized that digital occlusion analysis is a useful tool for assessing occlusal discrepancies and can be helpful during treatment planning and follow-up, especially for prosthetic therapy. Similar conclusions were presented by the authors of the work in the field of orthognathic therapy [21]. Traditional nondigital occlusal indicators, including articulating paper, foil, shim stock, and wax, provide only qualitative information about occlusal contacts [22,23]. They do not allow for measurement of contact force magnitude, intensity, or the duration of contact timing [24,25]. The T-Scan Novus enables dynamic measurement of OT as well as DT with accuracy, reliability, and reproducibility [10,26].

This study has certain limitations that should be acknowledged. It was conducted in a single center on a relatively limited number of patients, which means the results should be regarded as preliminary and confirmed in larger, multicenter studies. The inclusion criteria, while necessary to obtain a homogeneous sample and minimize confounding factors, excluded a broader spectrum of TMD patients, thereby narrowing clinical generalizability. Another limitation is that the follow-up was restricted to the immediate post-treatment period, and thus, the long-term stability of the clinical effects could not be assessed. In addition, although objective parameters such as disclusion time and occlusal force distribution were measured, patient-reported outcomes (pain intensity, functional improvement, and quality of life) were not included; integrating such measures in future studies would provide a more comprehensive clinical perspective.

Despite these limitations, the findings provide valuable preliminary evidence on the clinical usefulness of ICAGD enameloplasty combined with digital occlusion analysis, and highlight the need for further, long-term, randomized, and multicenter studies to validate and extend these observations.

## 5. Conclusions

In conclusion, our study highlights the value of selective enameloplasty using digital occlusal analysis in patients with temporomandibular joint dysfunction (TMD), emphasizing its role as an essential component of occlusal therapy. By eliminating occlusal interferences with digital analysis and achieving rapid posterior disclusion, well-planned enameloplasty helps reduce the load on the temporomandibular-duodenal joints and reduce muscle activity. These effects can lead to pain relief, improved mandibular mobility, and greater functional stability.

## Figures and Tables

**Table 1:** Baseline patients’ characteristics.

Gender, n (%) [Female]	73 (68.9%)
Center of force, n (%) [Appropriate]	49 (46.2%)
Premature occlusal contacts, n (%) [Yes]	75 (70.8%)
Age, IQR (95%CI) [years]	39 (33; 43)
Disclusion time before treatment, IQR (95%CI) [s]	0.80 (0.60; 0.90)
Disclusion time after treatment, IQR (95%CI) [s]	0.40 (0.30; 0.40)
Disclusion time difference before-after, IQR (95%CI) [s]	0.40 (0.20; 0.50)
Right site, IQR (95%CI)	51.50 (44.07; 57.64)
Left site, IQR (95%CI)	48.50 (42.36; 55.93)
Site difference right–left, IQR (95%CI)	3.00 (−11.86; 15.28)
Maximal bite force, IQR (95%CI) [N]	90.13 (84.58; 95.11)
Occlusion time before treatment IQR (95%CI) [s]	0.35 (0.26; 0.42)
Occlusion time after treatment, IQR (95%CI) [s]	0.35 (0.28; 0.42)
Occlusion time difference before-after, IQR (95%CI) [s]	−0.02 (−0.08; 0.08)

IQR—interquartile range, s—seconds.

**Table 2:** Studied population characteristic based on center of the force.

	Center of the Force		*p*-Value
	Appropriate n = 49	Not Appropriate n = 57	
Gender, n (%) [Female]	30 (61.2%)	43 (75.4%)	0.142
Age, IQR (95%CI) [years]	39 (33; 44)	39 (33; 42)	0.489
Disclusion time before treatment, IQR (95%CI) [s]	0.60 (0.50; 0.70)	0.90 (0.80; 0.90)	<0.001
Disclusion time after treatment, IQR (95%CI) [s]	0.30 (0.20; 0.40)	0.40 (0.40; 0.50)	<0.001
Disclusion time difference before-after, IQR (95%CI) [s]	0.29 (0.19; 0.39)	0.40 (0.40; 0.50)	<0.001
Right site, IQR (95%CI)	50.86 (46.93; 54.07)	57.64 (40.64; 61.50)	0.437
Left site, IQR (95%CI)	49.14 (45.93; 53.07)	42.36 (38.50; 59.36)	0.437
Site difference right–left, IQR (95%CI)	1.72 (−6.14; 8.14)	15.28 (−18.72; 23.00)	0.437
Maximal bite force, IQR (95%CI) [N]	90.59 (85.70; 94.52)	88.21 (83.73; 96.07)	0.470
Occlusion time before treatment IQR (95%CI) [s]	0.32 (0.23; 0.43)	0.36 (0.30; 0.42)	0.20
Occlusion time after treatment, IQR (95%CI) [s]	0.34 (0.27; 0.41)	0.35 (0.28; 0.44)	0.329
Occlusion time difference before-after, IQR (95%CI) [s]	−0.03 (−0.07; 0.07)	0.01 (−0.08; 0.08)	0.676
Premature occlusal contacts, n (%) [Yes]	18 (36.7%)	57 (100%)	<0.001

IQR—interquartile range.

## Data Availability

The data presented in this study are not publicly available due to patient confidentiality and ethical restrictions.
